# Poster Session II - A264 IL23R GENE POLYMORPHISM AS A PREDICTOR OF RESPONSE TO USTEKINUMAB (STELARA) IN PATIENTS WITH INFLAMMATORY BOWEL DISEASE

**DOI:** 10.1093/jcag/gwaf042.264

**Published:** 2026-02-13

**Authors:** Z Alhashimalsayed, A Wilson

**Affiliations:** Gastroenterology, Western University, London, ON, Canada; Medicine, Western University, London, ON, Canada

## Abstract

**Background:**

Ustekinumab, an interleukin (IL)-12/23 inhibitor, is widely used for treating inflammatory bowel disease (IBD). However, patient response to therapy varies considerably, potentially due to a myriad of factors. Genetic variables, in addition to clinical variables, are often not considered. Variation in key pathway genes, such as the *IL23R* gene, may affect drug response.

**Aims:**

To evaluate the association between the *IL23R 1142G>A* polymorphism and treatment outcomes in IBD patients receiving ustekinumab, including clinical remission, treatment durability, adverse events, and loss of response.

**Methods:**

A single-center, retrospective cohort study was conducted at Western University, including adult patients with Crohn’s disease (CD) or ulcerative colitis (UC) treated with Ustekinumab between 2014 and 2024. Patients were genotyped for *IL23R 1142G>A* and stratified into wild-type (GG) and variant carriers (GA). Clinical and laboratory data were extracted from electronic medical records. Outcomes included clinical remission (defined by the Harvey Bradshaw Index or partial Mayo score) at 6 and 12 months, treatment discontinuation, time to loss of response, treatment duration, and adverse events. Statistical comparisons used Mann–Whitney U and Fisher’s exact tests, while Kaplan–Meier analysis with log-rank testing assessed time-to-event outcomes.

**Results:**

Among the 95 eligible patients (92.6% CD, 7.4% UC), 82 (86%) carried the GG genotype, and 13 (14%) carried the GA variant. Baseline characteristics were similar between cohorts. Clinical remission at 12 months was achieved in 73.2% of patients with the GG genotype compared to 46.2% of those with GA (p = 0.06). Treatment discontinuation was significantly higher in GA patients (76.9%) than in GG (34.1%, p = 0.005), while the mean time to discontinuation was comparable between groups (2.63 ± 1.99 vs 2.20 ± 2.04 years, p = 0.51). Loss of response occurred in 84.6% of GA carriers and 72.0% of GG carriers (p = 0.50), with mean time to loss of response of 1.52 ± 1.58 and 1.50 ± 1.44 years, respectively (p = 0.75). Adverse events were more frequently observed in GA carriers (30.8%) compared to GG (3.7%, p = 0.006). Kaplan–Meier survival analysis demonstrated comparable treatment durability and time to loss of response between genotypes.

**Conclusions:**

The *IL23R 1142G>A* polymorphism is associated with higher treatment discontinuation and adverse event rates among a cohort of ustekinumab-treated IBD patients. Expansion of this work into larger cohorts will further delineate the role of *IL23R* variation in ustekinumab drug response.

**Funding Agencies:**

None

